# Study on Antibacterial Activities of *Croton macrostachyus* and *Pycnostachys abyssinica* Leaf Extracts Against Some Human Pathogens

**DOI:** 10.1155/tswj/9481587

**Published:** 2025-01-15

**Authors:** Getahun Yemata, Endalamaw Yihune, Yewulsew Kebede

**Affiliations:** Department of Biology, College of Science, Bahir Dar University, P. O. Box 79, Bahir Dar, Ethiopia

**Keywords:** bactericidal, bacteriostatic, *Croton macrostachyus*, inhibition zone, *Pycnostachys abyssinica*

## Abstract

The present study was aimed to verify the medicinal value of *Croton macrostachyus* and *Pycnostachys abyssinica* traditionally used to treat human and animal ailments in Ethiopia. Fresh leaves of these species were collected, dried under shade, and ground into fine powder. The extraction was carried out by the maceration method using methanol as a solvent. The compounds in the crude extract were further profiled by thin layer chromatography (TLC). The antibacterial activity of the compounds was evaluated using the agar well diffusion method. *C*. *macrostachyus* had a higher percentage extract yield (22.5%) than *P*. *abyssinica* (13.7%). The phytochemical screening showed more abundant phenolic compounds and tannins in the extract of *C*. *macrostachyus* and alkaloid, flavonoid, phenolic compounds, and tannins in the extract of *P*. *abyssinica*. Overall, *C*. *macrostachyus* produced twofold higher growth inhibition zone (24.0 ± 0.5–43.0 ± 1.0 mm) against the tested pathogens than *P*. *abyssinica* (7.0 ± 0.5–24.0 ± 0.3 mm). Among the bacteria, *Klebsiella pneumoniae* was found to be the most sensitive to compounds of *C*. *macrostachyus*. The lowest minimum inhibitory concentration (MIC) values (25 mg/mL) were obtained from compounds 1 and 3 of *C*. *macrostachyus* leaf extract against *Escherichia coli* and *K*. *pneumoniae* and compounds 2 and 3 of the same against *S*. *aureus*. Similarly, compound 1 of *P*. *abyssinica* leaf extract produced the lowest MIC (25 mg/mL) against *K*. *pneumoniae* and *Staphylococcus epidermidis* and compound 3 of the same species against *E*. *coli* and *K*. *pneumoniae*. All the profiled compounds of the two species had both bactericidal and bacteriostatic properties against the tested pathogens. The results of the present study revealed that the compounds of *C*. *macrostachyus* had strong antibacterial activity against all the tested pathogens, indicating the high potential of the compounds. However, further characterization and identification of the specific compounds for the development of biocide formulations are suggested.

## 1. Introduction

Plant secondary metabolites, also known as phytochemicals or natural products, are derivatives of primary metabolites synthesized to improve growth and survival under different environmental conditions [1]. Plants produce an enormous group of phytochemicals that explain their medicinal properties. This rich diversity of phytochemicals has been created due to evolutionary selection to improve the defense mechanism against a broad array of microorganisms and other enemies around plants [2]. These phytochemicals have curative properties, and man has been using these properties of plants since time immemorial. Man has discovered the curative properties of certain plants through instinct [3], trial and error [4], spiritual learning, and through watching how primates and different creatures use those plants [5].

According to the World Health Organization [6], 70%–95% of the world's population depends on traditional medicine for primary health care. Since a long time ago, phytochemicals have been widely used and documented in the pharmaceutical industry because of their extensive chemical and structural diversity and wide range of antimicrobial activity. Approximately, one-third of the drugs with the highest market exchange in the world are natural products or their derivatives often with ethnopharmacological upbringing [7].

Over time, interest in using natural products for the treatment of diseases has been increasing because conventional antibiotics become ineffective due to resistance development [8, 9], adverse side effects, and unaffordable price [8]. Drug resistance in most cases is caused by improper use of antibiotics in patients and extensive use of antibiotics in the animal industry. It is also caused by increase in the prescription of broad-spectrum antibiotics [9] and the preference of pharmaceutical companies to hunt antibacterial of microbial origin antibiotics, which are highly effective products that can be readily produced leading to rapid economic reward, but vulnerable to develop bacterial cross resistance [10]. Phytochemicals have been widely used in the pharmaceutical and food industry. In the food sector, the essential oils of various medicinal plants loaded to seed mucilages of different species have been found to inhibit microbial growth and lipid oxidation of stored meat. In support of this, Noshad et al. [11] have reported the antimicrobial activity of *Coriandrum sativum* seed and *Cuminum cyminum* essential oils against most foodborne pathogens. Essential oils of *Citrus paradise*, *Mentha pulegium*, *Citrus limon*, and *Cichorium intybus* loaded to *Lallemantia iberica*, *Ocimum basilicum*, *Plantago major*, and *Lepidium perfoliatum* seed mucilages, respectively, have been used as coatings of stored meat and inhibited microbial growth of *E*. *coli*, *S*. *aureus*, fungi, others, and lipid oxidation. Moreover, such coatings are found to preserve the color and sensory properties of the meat of different sources [12–15].

However, only a small fraction of approximately less than 1%–10% of the large diversity of plant species on Earth has been evaluated for the presence of different compounds and their medicinal properties [16, 17]. *Croton macrostachyus* Hochst. Ex Delile belongs to Euphorbiaceae family and is known by the vernacular name *Bisana* in Amharic language. *Pycnostachys abyssinica* Fresen is from the Lamiaceae family known for its aromatic oils. It is called *Tontana* in Wolaita and *Fanfo* in Sidama languages in Ethiopia. Traditionally, the two species are widely used to treat fungal and bacterial skin infections resulting in healing of wounds [18, 19], which stands as the main rationale for the present study. According to Megeresa et al. [20], sap of *C*. *macrostachyus* is used to stop bleeding, fired bark is used as mosquito repellent, juvenile leaves are used to treat ring worm, and powdered root mixed with *injera* (sour fermented pancake-like flatbread with a slightly spongy texture) is used to treat rabies. Sap is also applied topically to affected areas of the breast and skin to treat cancer [21]. Similarly, the leaf extract of *P*. *abyssinica* is used to treat eye disease [20] and skin cancer [21]. Different parts of *C*. *macrostachyus* have been found to possess active ingredients. Teugwa et al. [22]; Fetahi [23]; and Aylate et al. [24] have analyzed different parts of *C*. *macrostachyus* and found various phytochemicals. Similarly, Hussien, Hymete, and Rohloff [25] have reported alkaloids, saponins, flavonoids, and terpenoids in the extract of whole plant parts of *P*. *abyssinica*. The stem bark and essential oils of *C*. *macrostachyus* have demonstrated antigonorrheal activity against isolates of *Neisseria gonorrhoeae* [26] and antileishmanial activity [27], respectively. Likewise, leaf extract has shown antimicrobial activity against several clinical isolates of veterinary importance [28], antimycobacterial activity against *Mycobacterium tuberculosis* and *Mycobacterium bovis* strains [29], and antimalarial activity against *Plasmodium berghei* [30, 31]. The leaf extract of *P*. *abyssinica* has also exhibited antimicrobial activities against *S*. *aureus* [32]. However, scientific studies that validate the antimicrobial activity of *C*. *macrostachyus* and *P*. *abyssinica* leaf extracts against common human pathogens are scarce. Therefore, the objective of the present study was to evaluate the phytochemical composition and antibacterial activity of the methanol leaf extracts of the two species against some human pathogens.

## 2. Materials and Methods

### 2.1. Bacterial Strain

Clinical isolates of two Gram-positive bacteria (*Staphylococcus aureus* and *Staphylococcus epidermidis*) and two Gram-negative bacteria (*Escherichia coli* and *Klebsiella pneumoniae*) were obtained from the Amhara Public Health Institute (APHI) at Bahir Dar, Ethiopia. The strains were selected based on their high prevalence in the study area and their level of resistance to commonly used antibiotics. The findings of several studies conducted at Felege Hiwot Hospital, Bahir Dar, confirmed this. According to Moges et al. [33], *K*. *pneumonia*e (52.4%) was found to be the most prevalent followed by *E*. *coli* (12.4%). On the other hand, Belete et al. [34] identified *E*. *coli* as the most prevalent pathogen in the hospital. In the same year, Ayalew et al. [35] reported *S*. *aureus* (19.2%) as the predominant pathogen followed by *K*. *pneumoniae* (6.4%). Two years later, Abosse et al. [36] found out *S*. *aureus* as the most prevalent pathogen followed by *Klebsiella* species. The overall multidrug-resistant rates of *S*. *aureus*, *K*. *pneumoniae*, and *E*. *coli* isolates were found to be 88.9%, 92.6%, and 100%, respectively [35]. *S*. *epidermidis* has been found as the most important and frequent causative agent of health care–associated infections, increasing the costs of hospitalization, morbidity, and mortality in the recent years. *S*. *epidermidis* clinical isolates showed a high multidrug resistance that is not related to biofilm production [37]. Similarly, the proportion of methicillin resistance in *S*. *epidermidis* has been reported to be as high as 92% [38]. The strains were cultured overnight on the Mueller–Hinton agar (HiMedia Laboratories Pvt., Ltd., Mumbai, India). For antibacterial experiments, a single colony of each bacterial strain was inoculated from agar into 5 mL of tryptone soy broth (TSB) (HiMedia Laboratories Pvt., Ltd., Mumbai, India). The bacterial strains were incubated at 37°C.

### 2.2. Leaf Sample Collection

Fresh leaves of *C*. *macrostachyus* and *P*. *abyssinica* were collected from Bahir Dar city and Bure area, respectively. These plants were selected based on their traditional use in treating human ailments and follow-up of antimicrobial activity reports [39]. The leaf samples were washed with running tap water to remove dust and other debris. The leaf samples were dried under shade at room temperature. The dried leaf samples were ground to fine powder using an electrical grinder. The powder was sieved through a 0.5 mm mesh and stored in polyethylene bags. The bags were tightly closed and stored at room temperature. Plant specimens of the two species were collected, pressed, dried, and mounted. These specimens were used for identification using Flora of Ethiopia and Eritrea (Vol. 2 part 2 for *C*. *macrostachyus* and Vol. 5 for *P*. *abyssinica*). The specimens were also compared with authenticated specimens in the National Herbarium (ETH) at Addis Ababa University. An expert in plant taxonomy was involved in the process of identification. The voucher specimens were deposited at the National Herbarium, Addis Ababa University.

### 2.3. Extract Preparation

Separation of bioactive phytochemicals from leaf powders was carried out by the maceration method. The leaf powder of each species was added to a conical flask and poured with methanol in the ratio of 1:10 (w/v) before the maceration process (for every 1 g of leaf powder, 10 mL of solvent was added). The flask was tightly closed and placed on an orbital shaker. The mixture was continuously agitated for 72 h under room temperature condition at a speed of 200 rpm, until the soluble secondary metabolites are dissolved. After the end of the abovementioned period, the mixture was filtered with four layers of cheesecloth and cotton. The residue was squeezed into the receiving flask and finally discarded. The liquid extract was further passed through Whatman No. 1 filter paper. The solvent (methanol) was evaporated using a rotary evaporator. Methanol (99.8%) was used as a solvent in the process. The dried extracts of each species were stored at 4°C. Percentage yields of *C*. *macrostachyus* and *P*. *abyssinica* crude leaf extract were calculated according to Joshi and Kaur [40] as follows:

(1) $$\begin{array}{c} \text{Extract yield}\left(\%\right)=\frac{\text{Extract weight}\left(g\right)}{\text{Weight of plant powder}\left(g\right)}\times 100. \end{array}$$

#### 2.3.1. Component Separation of Extracts Through Thin Layer Chromatography (TLC)

**Figure 1:**
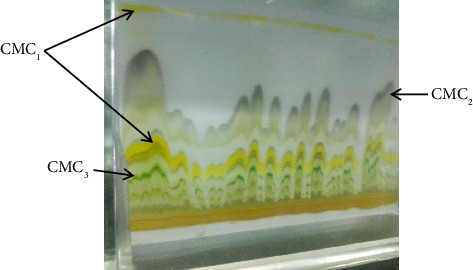
TLC compounds of methanol leaf extract of *C*. *macrostachyus* (CMC_1_, *C*. *macrostachyus* compound 1; CMC_2_, *C*. *macrostachyus* compound 2; CMC_3_, *C*. *macrostachyus* compound 3).

**Figure 2:**
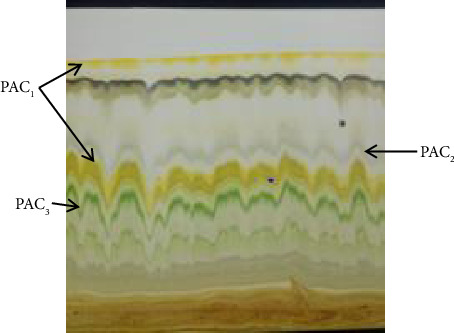
TLC compounds of methanol leaf extract of *P*. *abyssinica* (PAC_1_, *P*. *abyssinica* compound 1; PAC_2_, *P*. *abyssinica* compound 2; PAC_3_, *P*. *abyssinica* compound 3).

The TLC profiling of the extracts of *C*. *macrostachyus* and *P*. *abyssinica* was carried out using a glass TLC plate coated with silica gel. A light pencil line was drawn across the plate about 1 cm from the lower edge of the end of the TLC plates on which the extract was spotted. A separate TLC plate was used for each extract of the species. A capillary tube was used to spot the extracts, which were first dissolved in 1 mL of the macerating solvent (methanol). After filling the capillary by dipping the spot in the extracts, it was quickly touched on the cotton wool so that the spot did not diffuse. Spotted TLC plates were placed in a beaker with a watch glass on top, ensuring that the tank was cleaned and dried. The methanol–petroleum ether mixture in a 1:1 ratio was used as a developing solvent, poured into the beaker, and stirred. Tongs were used to place the TLC plate in the developing beaker carefully. The solvent rose to the TLC plate by capillary action. When the solvent reached within 1 cm from the top, the front of the plate was removed and the solvent was marked immediately with a pencil. The solvent was allowed to evaporate completely from the plate and visualized (Figures 1 and 2). After the extract dried on the TLC, the individual layers were scraped and collected separately. The collected extract was dissolved in the initial solvent and decanted to separate the extract from the silica gel. Finally, the solvent was evaporated and the residue extract was used for further qualitative analysis and antibacterial test.

#### 2.3.2. Preliminary Phytochemical Screening of TLC Components

Phytochemical analysis of the methanol extract of *C*. *macrostachyus* and *P*. *abyssinica* leaves was performed using standard procedures to determine the active constituents present in the TLC isolates. Tests for alkaloids, flavonoids, phenols, tannins, and terpenoids were performed following the methods of Pandey et al. [41].

##### 2.3.2.1. Test for Alkaloids

Dragendorff's test: Approximately 1 mL of each species extract was taken and stirred properly with the addition of 1 mL of the Dragendorff reagent. A reddish-brown precipitate confirmed the presence of alkaloids.

##### 2.3.2.2. Test for Flavonoids

Alkaline reagent test: The extract solution (3 mL) of each species was treated with a few drops of sodium hydroxide solution. The presence of flavonoid was identified by the formation of an intense yellow color that becomes colorless with the addition of dilute acetic acid.

##### 2.3.2.3. Test for Tannins

Ferric chloride test: Extract solution (3 mL) was taken and a few drops of 0.1% ferric chloride solution were added and allowed to diffuse for a few minutes. Tannin's presence was identified by the formation of brownish green or blue-black color.

##### 2.3.2.4. Test for Terpenoids

Salkowski test: Extract solution (2 mL) was treated with 2 mL of chloroform and filtered. The filtrate was treated with few drops of concentrated H_2_SO_4_, shaken, and allowed to stand. The presence of terpenoids was confirmed by formation of yellow color at the lower layer.

##### 2.3.2.5. Test for Phenols

The methanol extract (2 mL) was put in a test tube and treated with a few drops of 2% FeCl_3_. Bluish green coloration indicated the presence of phenols.

### 2.4. Evaluation of the Antibacterial Activity of Leaf Extracts

The bacterial suspension was prepared from a 24 h old pure culture of *E*. *coli*, *K*. *pneumoniae*, *S*. *aureus*, and *S*. *epidermidis*. Sterilized distilled water was used to prepare the suspension. The optical density of the suspension was adjusted to 0.132 at 600 nm using a spectrophotometer. This is equivalent to 0.5 McFarland turbidity standards. The number of bacterial populations at this turbidity standard is 1.5 × 10^8^ CFU/mL.

The in vitro antibacterial activity of TLC isolates of the studied plant species was evaluated using the agar well diffusion method as described by Taye et al. [42]. The plate filled with the Mueller–Hinton agar was inoculated by swabbing 100 μL of standardized bacterial suspension of the tested pathogens over the entire surface. Then, wells/holes of 4 mm in diameter were punched aseptically with sterile cork borer on each plate. The test concentrations (100, 50, and 25 mg/mL) of each species extract were prepared using the original solvent and 100 μL of each test extract concentration was introduced into the agar well. The agar wells were situated 2 mm far apart. Each plate was incubated at 37°C for 24–48 h. The minimum and maximum test concentrations were determined based on the research reports of previous studies and the feasibility of the concentrations for further drug development. Equal volume of methanol and 30 μg/mL of tetracycline antibiotic were introduced into the wells of negative control and standard plates, respectively. Each plate was labeled according to the treatments (TLC isolates, bacterial species, standard, and negative control). The antibacterial extract diffuses in the Mueller–Hinton growth medium and inhibits the growth of the tested pathogens. The inhibition zone diameter around each well was measured in mm with a transparent ruler. The assay was carried out in three replications for each TLC isolate. The interpretation of the antibacterial characteristics of plant extracts was carried out as described in Adithepchaikarn et al. [43]. Inhibition zones > 15 mm were classified as strong activity, 10–15 mm as moderate activity, and < 10 mm as weak activity.

#### 2.4.1. Determination of Minimum Inhibitory Concentration (MIC) and Minimum Bactericidal Concentration (MBC)

The MIC of the TLC profiled compounds was determined using a broth dilution method as described by Balouiri, Sadiki, and Ibnsouda [44]. The stock solution (200 mg/mL) of each extract of the two plant species was serially diluted to yield 100 mg/mL, 50 mg/mL, and 25 mg/mL. From each dilution, 100 μL of extract was dispensed to a tube containing 2 mL broth. Each tube was inoculated with a bacterial suspension prepared from broth and consisted of 1.5 × 10^6^ CFU/mL. The content of each tube was well mixed and incubated at 37°C. In parallel, tubes with equal volume of broth without extract served as positive control. The amount of growth in each tube was compared with that in the positive control as detected by unaided eye. The MIC was recorded as the lowest concentration of the extract that either completely inhibited growth or inhibited 80% of growth as compared to the positive control. Similar to MIC, MBC was determined using the broth dilution method by adding various concentrations of extracts above MIC in the Mueller–Hinton broth. Then, the tubes were inoculated with the tested pathogens and incubated at 37°C for 24–48 h. After the completion of the incubation period, the tubes, which showed no visible bacterial growth, were subcultured on extract-free Mueller–Hinton agar plates and incubated at 37°C for 24–48 h. Therefore, the lowest extract concentration that inhibited the growth of the pathogen in the medium was considered as the MBC. Each experiment was carried out in triplicate. Moreover, the mechanism of antibiosis (bacteriostatic or bactericidal) was determined according to Shanmughapriya et al. [45] as MBC/MIC ratio. When the ratio of MBC/MIC is ≤ 2, the TLC profiled compounds were considered as bactericidal otherwise as bacteriostatic. If the ratio is 16, the TLC profiled compounds were considered as ineffective.

### 2.5. Data Analysis

Data were analyzed using SPSS software Version 23. Statistical differences in the mean zones of inhibition for individual bacterium and differences in the susceptibility of the test microorganisms were analyzed using ANOVA followed by Tukey's post hoc test at a significance level of *p* ≤ 0.05.

## 3. Results

### 3.1. Extract Yield

The result of the present study showed variation in percentage extract yield between the studied medicinal plants species. *C*. *macrostachyus* (22.5%) had a higher percentage extract yield than *P*. *abyssinica* (13.7%).

### 3.2. Preliminary Screening of Phytochemicals

The preliminary phytochemical screening of the TLC profiled compounds of *C*. *macrostachyus* and *P*. *abyssinica* revealed variation both in concentration and in diversity of secondary metabolites. *C*. *macrostachyus* compound 3 (CMC_3_) had more abundant phenols and tannins and abundant alkaloids and flavonoids. It also consisted of all secondary metabolites (Table 1). *C*. *macrostachyus* compound 2 (CMC_2_) was found to have the lowest concentration and diversity (Table 1). Similarly, more abundant alkaloid, flavonoid, phenols, and tannins were recorded in *P*. *abyssinica* compound 3 (PAC_3_) (Table 1).

### 3.3. Antibacterial Activity of TLC Profiled Compounds

The results of the present study demonstrated a significantly different antibacterial activity between the TLC profiled compounds of *C*. *macrostachyus* and *P*. *abyssinica* against the tested human pathogens (Tables 2 and 3). Comparatively, *C*. *macrostachyus* TLC profiled compounds showed remarkably higher antibacterial activity than *P*. *abyssinica* compounds. For all profiled compounds evaluated, a concentration-dependent increase in the antibacterial activity was observed. Moreover, the study bacteria exhibited variable susceptibility to the profiled compounds of the two medicinal plant species (Tables 2 and 3).

TLC profiled compounds of *C*. *macrostachyus* displayed significantly variable antibacterial activity against the tested pathogens at *p* ≤ 0.05 (Table 2). A significantly higher antibacterial activity was recorded by the profiled compounds of *C*. *macrostachyus* (CMC_1_, CMC_2_, and CMC_3_) against the tested pathogens as compared to the standard antibiotic (tetracycline) at *p* < 0.05. The compounds exhibited growth inhibition zone ranging from 24.0 ± 0.5 to 43.0 ± 1.0 mm against all the tested pathogens. CMC_3_ had a significantly higher potency than the other *C*. *macrostachyus* compounds (CMC_1_ and CMC_2_) and showed the widest growth inhibition zone (43 mm) at 100 mg/mL test concentration against *K*. *pneumoniae* (Table 2). In addition, a statistically significant variation was recorded between test concentrations under each TLC profiled compound against all the tested pathogens. Test concentrations (50 and 100 mg/mL) of all TLC profiled compounds had significantly greater antibacterial activity than the standard antibiotic used. The result of the antibacterial susceptibility assay revealed that *K*. *pneumoniae* is relatively the sensitive species to TLC profiled compounds of *C*. *macrostachyus* (Table 2).

*The P*. *abyssinica* TLC profiled compounds exhibited antibacterial activity against the tested pathogens. However, the differences were significantly lower than the antibacterial activity of the standard antibiotic at *p* < 0.05 (Table 3). The TLC profiled compounds showed antibacterial activity ranging from 7.0 ± 0.5 mm to 24.3 ± 0.3 mm in inhibition zone diameter. In most, the differences in antibacterial activity between the test concentrations of TLC profiled compounds were statistically nonsignificant (Table 3). The widest growth inhibition zone (24.3 ± 0.3 mm) recorded by TLC profiled *P*. *abyssinica* compound 3 (PAC_3_) against *S*. *epidermidis* was lower almost by half as compared to CMC_3_ (Table 3). *E*. *coli* was found to be relatively resistant to *P*. *abyssinica* compounds.

### 3.4. MIC and MBC of TLC Profiled Compounds

**Figure 3:**
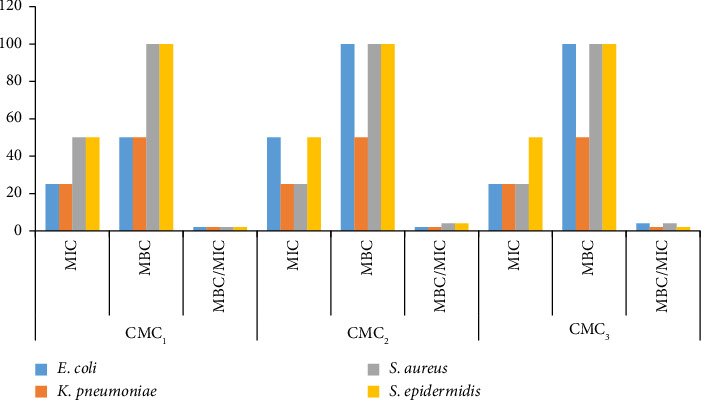
Minimum inhibitory, bactericidal concentrations and MIC indices of TLC profiled compounds of *C*. *macrostachyus* (CMC_1_, *C*. *macrostachyus* compound 1; CMC_2_, *C*. *macrostachyus* compound 2; CMC_3_, *C*. *macrostachyus* compound 3).

Different MIC and MBC values were obtained from the different TLC profiled compounds of the two species against *E*. *coli*, *K*. *pneumoniae*, *S*. *aureus*, *and S*. *epidermidis* (Figure 3). The lowest MIC (25 mg/mL) was obtained from CMC_1_ and CMC_3_ against *E*. *coli* and *K*. *pneumoniae*. Similarly, the lowest MIC was obtained from CMC_2_ and CMC_3_ against *S*. *aureus*. The lowest MBC (50 mg/mL) value was recorded for *K*. *pneumoniae* by CMC_1_, CMC_2_, and CMC_3_. The same was documented for *E*. *coli* by CMC_1_ (Figure 3).

**Figure 4:**
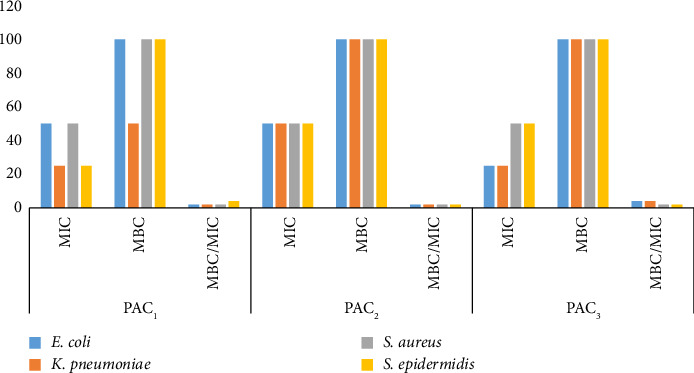
Minimum inhibitory, bactericidal concentrations and MIC indices of TLC profiled compounds of *P*. *abyssinica* crude leaf extract. PAC_1_, *P*. *abyssinica* compound 1; PAC_2_, *P*. *abyssinica* compound 2; PAC_3_, *P*. *abyssinica* compound 3.

Similarly, the MIC and MBC of *P*. *abyssinica* showed almost the same trend as those of *C*. *macrostachyus*. The highest MIC (50 mg/mL) values were recorded by PAC_3_ against *E*. *coli* and *S*. *aureus*, PAC_1_ against *S*. *aureus* and *S*. *epidermidis*, and by PAC_2_ against all the test pathogens (Figure 4). PAC_3_ and PAC_1_ were potent in that they inhibited the growth of the tested bacteria at relatively low concentration as compared to others. Accordingly, PAC_1_ had the lowest MIC (25 mg/mL) against *K*. *pneumoniae* and *S*. *epidermidis*. PAC_3_ showed the lowest MIC against *E*. *coli* and *K*. *pneumoniae*. All compounds of *P*. *abyssinica* (PAC_1_, PAC_2_, and PAC_3_) had a higher MBC value against all the tested pathogens except PAC_1_ against *K*. *pneumoniae* (Figure 4).

According to the MBC/MIC value, all the TLC profiled compounds of the two species had both bactericidal and bacteriostatic properties against the tested pathogens. CMC1, PAC2, and all other compounds except S. epidermidis for PAC1 killed the bacteria (Figures 3 and 4). The remaining compounds stopped the bacteria from growing. None of the tested compounds had no effect on the bacteria (Figures 3 and 4).

## 4. Discussion

The results of the present study demonstrated higher percentage extract yield in *C*. *macrostachyus* than *P*. *abyssinica* [46]. This variation might be due the difference in solubility of the leaf powder of the two species. The leaf powder of *C*. *macrostachyus* had higher extractable compounds in methanol than *P*. *abyssinica*. Evidently, the solubility of phenolic compounds is mostly influenced by the nature of the solvent and its polarity. Higher extract yield has been found from polar solvents such as methanol [47]. Different extract yield values are reported by various authors for the two species. Significantly lower extract yield has been found from the leaf [22, 32] and bark [48] samples of *C*. *macrostachyus*. This might be due to the difference in methodology including the solvent to sample ratio and part of the plant sampled. In the contrary, Hussien et al. [25] have reported higher extract yield than the present study, which might be due to the variation in the extraction process: gradient solvent extraction versus maceration method. The higher extract yield can be taken as one form of standardization parameter and be indicative of the amount of active ingredient that could be considered in other possible production processes.

The TLC profiled compounds of *C*. *macrostachyus* (CMC_1_, CMC_2_, and CMC_3_) and *P*. *abyssinica* (PAC_1_, PAC_2_, and PAC_3_) demonstrated variation in concentration and almost similar diversity of secondary metabolites. Regardless of the variation, less to more abundant amounts of alkaloid and phenolic compounds were recorded in the methanol leaf extract of *C*. *macrostachyus*. Similar findings have been reported by Teugwa et al. [22]; Aylate et al. [24]; Kumudin et al. [49]; and Fetahi [23]. According to Aylate et al. [24], the methanol leaf extract of *C*. *macrostachyus* is found to possess less abundant alkaloids and phenolic compound species. This slight variation might be due to climatic and soil factor differences in the sites from where the samples were collected. In line with the present study, Dindamo et al. [50] have found alkaloids, tannins, and terpenoids in the ethanol leaf extract of *C*. *macrostachyus*. On the other hand, the methanol extract of *P*. *abyssinica* had less to more abundant amount of alkaloids, phenolic compound species, and terpenoids. This agrees with the research results of Yemata and Fetene [51]. The findings of the present study in general revealed the presence of three compounds in each leaf extract of each medicinal plant species with variable concentration and diversity of secondary metabolites responsible for antibacterial activity. In this regard, the leaf extract of *C*. *macrostachyus* had strong antibacterial activity as growth inhibition zones at all test concentrations are ≥ 15 mm against the tested human pathogens [43]. This is by far greater than the reports of Obey et al. [52]; Romha et al. [53]; Aylate et al. [24]; and Firomsa et al. [54]. According to Firomsa et al. [54] and Fetahi [23], the methanol leaf extract of *C*. *macrostachyus* has produced 4.4 ± 0.2 mm and 13.3 ± 2.3 mm zone of inhibition against *E*. *coli* at 50 mg/mL and 250 mg/mL, respectively. The leaf extract of the same species has shown 12.8 mm and 4.4 mm zone of inhibition against *S*. *aureus* at 250 and 200 mg/mL test concentrations, respectively [23, 53]. Similar results of methanol leaf extract have been reported by Aylate et al. [24] against *E*. *coli* (9.3 mm) and *S*. *aureus* (21 mm) at 200 mg/mL test concentration. A corresponding growth inhibition zone has been found by the methanol bark extract of *C*. *macrostachyus* against *E*. *coli* (9 mm) and *K*. *pneumoniae* (14.9 mm) [52]. However, the TLC profiled compounds of *C*. *macrostachyus* had stronger antibacterial activity as compared to the findings of previous studies. In support of this, Dindamo et al. [50] have found 8.67 and 9.83 mm growth inhibition zone of *E*. *coli* and *S*. *aureus*, respectively, due to 100 mg/mL ethanol leaf extract of *C*. *macrostachyus*. The observed variation can be explained by the abundance and diversity of phenolic compounds, alkaloids, and terpenoid species in the TLC profiled compounds with different modes of action and synergistic effect.

According to the interpretation of the antibacterial activity of plant extracts, the *P*. *abyssinica* TLC profiled compounds had weak and moderate activities (< 10 mm and 10–15 mm zone of inhibition, respectively) at 25 and 50 mg/mL against all pathogens and strong activity at 100 mg/mL against *K*. *pneumoniae*, *S*. *aureus*, and *S*. *epidermidis* [43]. This agrees with the findings of Gebre-Mariam et al. [32] and Hussien, Hymete, and Rohloff [25] who have described weak to moderate activities of the methanol leaf extract of *P*. *abyssinica* against *S*. *aureus*.

The MIC and MBC values of the studied medicinal plants indicated a moderate antibacterial activity against the tested pathogens. Both higher and lower MIC values have been disclosed by Obey et al. [52]; Romha et al. [53]; and Aylate et al. [24]. Obey et al. [52] have found a higher MIC value of *C*. *macrostachyus* methanol bark extract against *E*. *coli* (250 mg/mL) and *K*. *pneumoniae* (500 mg/mL). On the other hand, Aylate et al. [24] and Romha et al. [53] have reported lower MIC values against *E*. *coli* and *S*. *aureus*. Similar lower MIC values of the *P*. *abyssinica* extract have been reported by Hussien, Hymete, and Rohloff [25] and Messele et al. [32] against *E*. *coli* and *S*. *aureus*. This difference in MIC might be attributed to the variation in the extraction method and plant parts. However, according to Kuete [55], the MIC value of the present study is weak. Kuete [55] categorized the activity of plant extracts as significant when MIC < 100 μg/mL, moderate when 100 < MIC ≤ 625 μg/mL, and weak when MIC > 625 μg/mL. Furthermore, the TLC profiled compounds of the studied species were found to have bactericidal and bacteriostatic effect. This agrees with the findings of Bakari et al. [56] who have described the bactericide role against *K*. *pneumoniae* and bacteriostatic against *E*. *coli* and *S*. *aureus*. Nonetheless, the antibiosis mechanisms of the TLC profiled compounds of the two species were noted to be bactericidal, which is in line with the reports of Thomas et al. [57] and Mogana et al. [58]. The bacteriostatic and bactericidal properties of the extracts provide a promising approach to combating antibiotic resistance. This is because plant extracts contain a variety of bioactive compounds with diverse and new modes of actions compared to antibiotics. Moreover, plant extracts work either individually and/or synergistically against pathogen, which in turn maximizes their efficiency [59].

In addition to the concentration and diversity of secondary metabolites in the TLC profiled compounds of the medicinal plants, the strong and moderate antibacterial activity can be explained by the synergistic effect and different modes of actions of the metabolites. Accordingly, phenolic compounds disrupt energy production due to inhibition by the oxidized products through reaction with sulfhydryl groups or through more nonspecific interactions with the proteins [7] and by inhibiting bacterial fatty acid synthesis [60]. Terpenoids hinder the growth of bacteria by disrupting cell membrane integrity [9, 61]. Similarly, flavonoids and tannins target the bacterial envelope, inactivating the membrane through interaction with membrane proteins and microbial adhesins [9]. Furthermore, alkaloids inhibit efflux pump [62], intercalate with DNA, and inhibit protein synthesis, which subsequently results in impaired cell division and cell death [9, 63].

The significantly higher antibacterial activity of compounds of *C*. *macrostachyus* compared with *P*. *abyssinica* against the tested pathogens can be explained by the difference in the ecology of the two species. Ecologically, *P*. *abyssinica* grows in wet montane forests and is also planted in hedges, while *C*. *macrostachyus* is a widespread tree that grows commonly in secondary forests, and at the edges of forests especially at forest edges and along the road with high potential exposure to different environmental stress factors, such as light and drought. Evidently, an elevated content of phenolic compounds, flavonoids, and photosynthetic pigments has been reported in several medicinal plants under severe drought conditions [64]. Exposure of plants to UV radiation especially UV-B has also been noted to instigate the biosynthesis of alkaloids, total phenolics, anthocyanins, carotenoids, flavonoids, lignin, tannins, and saponins [65]. Such augmented productions of secondary metabolites in plants are meant to induce tolerance to environmental stress factors [66]. Moreover, the difference in antibacterial activity between the two species might be caused by the age of the sampled leaves, harvesting season, and growth stage as the biosynthesis of some secondary metabolites begins as early as the first cotyledon and others in matured leaves in the later stages of development [67].

## 5. Conclusions

The result of the present study showed variation in percent extract yield between the studied medicinal plants. *C*. *macrostachyus* had a higher percentage extract yield than *P*. *abyssinica*. The extracts of *C*. *macrostachyus* and *P*. *abyssinica* contained phenols, tannins, flavonoids, and alkaloids. The methanol TLC isolates of the medicinal plant species showed different antibacterial activity attributed to the concentration and diversity of secondary metabolites. Although not quantified, extracts with abundant amount and diversity of secondary metabolites had higher antibacterial activity. The compounds profiled with TLC from both species had bacteriostatic and bactericidal characteristics. CMC_1_, PAC_1_ (except for *S*. *epidermidis*), and PAC_2_ showed bactericidal role against all the tested pathogens. Generally, CMC_1_, CMC_3_, and PAC_3_ had higher antibacterial activity, which could be potential candidates for further studies and development of biocide formulations to control pathogenic bacteria. This study is important as it generated baseline data for further studies. Further studies are needed about the quantification of phytochemicals and characterization and identification of the specific compounds in the studied medicinal plants [67].

## Figures and Tables

**Table 1:** Preliminary analysis results of TLC profiled compounds of methanol leaf extracts of *C*. *macrostachyus* and *P*. *abyssinica*.

Plant species	TLC profiled compounds	Phytochemicals				
		Alkaloids	Flavonoids	Phenols	Tannins	Terpenoids
*C*. *macrostachyus*	Compound 1	++	++	++	++	−
	Compound 2	+	++	+	+	−
	Compound 3	++	++	+++	+++	+

*P*. *abyssinica*	Compound 1	++	++	++	++	+
	Compound 2	+	+	+	+	+
	Compound 3	+++	+++	+++	+++	++

*Note:* +, less abundant; ++, abundant; +++, more abundant; −, absent.

**Table 2:** Growth inhibition zones (mm) of TLC profiled compounds of C. *macrostachyus* against human pathogenic bacterial strains.

**Bacterial strains**	**Test concentrations of *C. macrostachyus* compounds (mg/mL)**									
	CM**C**_1_			CM**C**_2_			CM**C**_3_			
	**25**	**50**	**100**	**25**	**50**	**100**	**25**	**50**	**100**	**Tetracycline**

*E*. *coli*	27.3 ± 0.3^aA^	30.66 ± 0.6^bA^	35.33 ± 1.5^cB^	31 ± 0.6^dA^	33.6 ± 1.9^eB^	36.33 ± 0.9^fA^	34.66 ± 1.5^gB^	37 ± 1.15^hA^	37 ± 1.5^hA^	30 ± 0.0^aA^
*K*. *pneumoniae*	38 ± 1.2^aB^	40 ± 0.0^bB^	42.66 ± 1.2^cA^	34 ± 1.2^dA^	41.3 ± 0.9^eC^	42 ± 0.6^cB^	34.6 ± 0.9^dB^	39.6 ± 1.4^eA^	43 ± 1.0^fB^	30 ± 0.0^gA^
*S*. *aureus*	25 ± 0.5^aA^	31 ± 0.5^bA^	32 ± 1.0^cAC^	33 ± 1.5^dA^	26.3 ± 0.8^dD^	35.6 ± 1.2^eC^	22.3 ± 0.9^fB^	35 ± 0.5^eA^	34.3 ± 0.3^gC^	30 ± 0.0^aA^
*S*. *epidermidis*	24 ± 0.5^aC^	34.3 ± 0.3^bC^	30.6 ± 0.8^cA^	35.3 ± 1.7^dA^	36 ± 2.0^eB^	37.6 ± 1.2^fD^	31.6 ± 1.2^gB^	32 ± 1.8^gB^	33 ± 1.0^hD^	30 ± 0.0^cA^

*Note:* Values with similar small letters in a row and capital letters in a column showed statistically insignificant difference between means at *p* ≤ 0.05.

Abbreviations: CMC_1_, *C*. *macrostachyus* compound 1; CMC_2_, *C*. *macrostachyus* compound 2; CMC_3_, *C*. *macrostachyus* compound 3.

**Table 3:** Growth inhibition zones (mm) of TLC profiled compounds of *P*. *abyssinica* against human pathogenic bacterial strains.

**Bacterial strains**	**Concentration of TLC profiled compounds of *P. abyssinica* crude leaf extract (mg/mL)**									
	PA**C**_1_			PA**C**_2_			PA**C**_3_			
	**25**	**50**	**100**	**25**	**50**	**100**	**25**	**50**	**100**	**Tetracycline**

*E*. *coli*	7 ± 0.6^aA^	8.6 ± 0.3^bA^	10.3 ± 0.3^cA^	10.3 ± 0.3^cA^	10 ± 0.0^cA^	14 ± 0.6^dA^	7 ± 0.6^aA^	8.6 ± 0.6^bA^	10.3 ± 0.3^cA^	30 ± 0.0^e^
*K*. *pneumoniae*	10.3 ± 0.3^aB^	11 ± 0.6^dB^	16 ± 0.6^bB^	10.3 ± 0.3^aA^	10 ± 0.0^aA^	22.3 ± 0.8^cB^	10.6 ± 0.3^cB^	11.6 ± 0.3^dB^	22 ± 0.6^eB^	30 ± 0.0^f^
*S*. *aureus*	8.6 ± 0.3^aAB^	9.3 ± 0.3^aAB^	19 ± 0.6^bC^	9.6 ± 0.3^aA^	11.3 ± 0.3^aA^	18.6 ± 0.6^bC^	8.6 ± 0.3^aA^	10.6 ± 0.3^aC^	21.6 ± 0.6^cB^	30 ± 0.0^d^
*S*. *epidermidis*	9.3 ± 0.3^aCB^	11 ± 0.6^bB^	17 ± 0.6^cD^	11.6 ± 0.3^bA^	13 ± 0.6^bB^	20 ± 0.6^cD^	9.3 ± 0.3^aC^	10.3 ± 0.3^bC^	24.3 ± 0.3^dC^	30 ± 0.0^f^

*Note:* Values with similar small letters in a row and capital letters in a column show a statistically nonsignificant difference between means at *p* < 0.05.

Abbreviations: PAC1, *P. abyssinica* compound 1; PAC2, *P. abyssinica* compound 2; PAC3, *P. abyssinica* compound 3.

## Data Availability

The data used to support the findings of this study are available on reasonable request to the corresponding author.
